# Analyzing coordination structures for effective humanitarian relief operations

**DOI:** 10.1038/s41598-025-33588-1

**Published:** 2026-01-11

**Authors:** Iman Parsa, Mahyar Eftekhar, Scott Webster, Luk N. Van Wassenhove

**Affiliations:** 1 Department of Entrepreneurship, Innovation and Technology, Stockholm School of Economics, Stockholm, Sweden; 2 W.P. Carey School of Business, Arizona State University, Tempe, Arizona, US; 3 Technology and Operations Management, INSEAD, Fontainebleau, France

**Keywords:** Humanitarian response, Horizontal coordination, Coordinated decision-making, Game theory, Engineering, Environmental social sciences

## Abstract

Despite significant efforts by humanitarian actors, initiatives, and donors to improve coordination among humanitarian organizations during disaster response, the challenge of insufficient coordination persists. Drawing on practical considerations, we develop a stylized non-cooperative game-theoretical model to examine the coordination dynamics between large international and small local humanitarian organizations in the aftermath of a disaster, comparing both *pooled* and *partitioned* coordination models. Our findings reveal that bureaucratic delays commonly associated with coordination efforts not only deter collaboration but can also result in coordination levels that are detrimental to overall relief system performance. This analysis underscores the importance of rethinking coordination structures to better reflect the specific context of relief efforts. In alignment with calls for increased localization, we demonstrate that an efficiently designed partitioned coordination model outperforms a pooled model, which often marginalizes smaller local actors. This partitioned approach proves particularly effective in emergency settings, bringing actors’ decisions closer to the optimal outcome for the entire system.

## Introduction

Between 2000 and 2023, natural hazards affected an average of 190 million people annually^1^. The effective delivery of aid to these populations relies heavily on coordination among non-governmental humanitarian organizations (HOs). In disaster response, coordination is critical due to the simultaneous surge in demand and the limited resources available to HOs^2^. It plays a vital role in enhancing the efficiency of relief efforts by, for example, preventing duplication of activities and ensuring that no individuals are overlooked. Consequently, coordination improves the allocation and utilization of HOs’ constrained resources, enabling them to better respond to shifting needs^3^. Despite the introduction of frameworks such as the UN’s *Cluster Approach *and advocacy from major donors like the European Civil Protection and Humanitarian Aid Operations (ECHO), significant challenges remain^4^. For instance, the response to the recent Türkiye-Syria earthquakes was criticized for its lack of effective coordination^5^. In particular, existing coordination models often marginalize smaller local HOs, despite their crucial role in rapid response^6,7^. During the Ebola outbreak in West Africa, the exclusion of small HOs with local knowledge and expertise contributed to sub-optimal decisions and ineffective coordination^8^. This issue persists despite the sector’s ongoing commitment to localization, which explicitly seeks to include local actors, as seen in initiatives like the Grand Bargain^9^.

This paper focuses on coordination between non-governmental HOs within a relief system responding to a specific disaster, known as *horizontal coordination*. We define coordination as *coordinated decision-making *that seeks to align the operations of HOs^3^. This concept extends beyond mere information exchange, which is essential for coordination but does not by itself guarantee aligned operations^10,11^. Our definition deliberately excludes resource sharing, as it requires a higher level of collaboration and introduces significant additional challenges^12^. These challenges stem from the need for HOs to maintain operational independence, adhere to donor restrictions, and manage other constraints. Similarly, this study does not examine disaster response in conflict-affected regions, as the specific challenges in those contexts, such as access constraints, security risks, and the involvement of governmental or military actors, lead to different dynamics.

Despite its advantages, coordination often involves time-consuming processes and bureaucratic procedures that can delay HOs’ operations^13^. This is particularly problematic in urgent disaster response, where urgency makes time one of the most critical and limited resources^14^. Moreover, resource scarcity fuels competition among HOs for sustainable funding, as their visibility and performance during the response impact their long-term survival^15^. Competition for media attention, for instance, has been linked to failures in the Haiti response^16^. Ultimately, the benefits of coordination depend on the effectiveness of the coordination model. Poorly designed systems increase inefficiencies and time burden that discourage participation. There is growing recognition of the need for decentralization and development of local coordination structures, i.e., localization^17^.

These conflicting dynamics challenge the assumption that more coordination always leads to better outcomes, yet the trade-offs are often underexplored. The prevailing belief that all HOs should participate in coordination persists. This paper addresses this gap by analyzing these tensions within the disaster response context. From a high-level perspective, we examine critical factors such as response urgency, bureaucratic obstacles, and coordination structures. Our primary objective is to identify conditions under which increased participation of HOs improves relief system performance. Additionally, we compare coordination levels and resulting performance in both *pooled* and *partitioned* models.

**Fig. 1:**
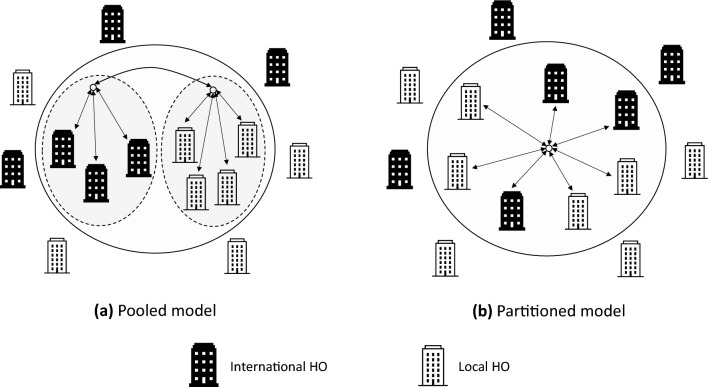
Illustration of coordination models. In the *pooled* model, all HOs form a single coalition, directly coordinating and communicating on a shared platform. In contrast, the *partitioned* model separates local and international HOs into sub-coalitions, which are loosely coordinated at a higher level.

A *pooled *coordination model unites international and local HOs into a single coalition, aiming to leverage their complementary strengths. International HOs contribute extensive experience across diverse crises and possess the capacity for large-scale planning and execution. However, localization initiatives consistently emphasize that international HOs often lack the critical contextual knowledge that local HOs provide despite their operational capacity. This contextual information includes field conditions, local supplies, the needs of the affected population, and cultural nuances. Distinctions between international and local HOs can hinder participation and dilute the diversity advantages of a pooled model. Critiques of over-centralization and the limited involvement of local HOs have led to the exploration of alternative approaches^6^. In a *partitioned*model, small local and large international HOs form separate sub-coalitions, which are coordinated at a higher level without requiring direct coordination between all actors. For instance, sub-coalitions can allocate geographical areas based on aggregated demand estimates and capacities, aligning with the emphasis on decentralized structures and localized responses^17^. A practical example is the South Sudan NGO Forum, where national and international forums operate separately, with steering committees coordinating at a higher level. Figure 1 illustrates these two models.

Given the strength of game theory in analyzing interconnected decisions made by multiple actors^18^, we develop a game-theoretical model to study HOs’ coordination decisions. This approach allows us to assess the problem from the perspectives of both HOs and a central coordinator, e.g., the Emergency Response Coordinator, who aims to optimize system performance. Our model is inspired by the UN Logistics Cluster, which plays a prominent role in large-scale disaster settings by facilitating coordination among non-governmental HOs in the field. It is one of the eleven clusters that make up the UN’s humanitarian coordination system^19^. To ensure the model reflects real-world dynamics, we conducted a field study involving unstructured interviews with executives from UN agencies, as well as both international and local HOs, within and outside the UN Logistics Cluster. This field study aimed to identify key incentives and barriers to coordination, as well as to validate the assumptions and main insights of the paper. We construct a generic utility function, informed by the literature and our field study. The utility function captures key incentives and disincentives for coordination, particularly in terms of operational performance metrics such as response speed (timeliness)^14^ and demand coverage^3^. It also accounts for HOs’ anticipated income, which reflects their financial sustainability concerns, including public donations driven by media exposure and grants from major donors tied to operational outcomes^15^.

To analyze these dynamics, we employ a sequential non-cooperative game involving both local and international non-governmental HOs, examining their optimal (rational) decisions. We explore scenarios in which HOs are either forward-looking, anticipating the decisions of subsequent players, or myopic, ignoring these future decisions. The outcomes are compared with decisions that maximize relief system performance, allowing us to evaluate pooled and partitioned coordination models. We compare the two models based on three key criteria: (1) coalition size, i.e., the number of participating HOs in the coalition; (2) relief system performance, i.e., the total utility across all HOs that do and do not join the coalition; and (3) alignment with the social optimal solution, i.e., the gap between the equilibrium and optimal coalition sizes.

## Results and contributions

Our findings emphasize the importance of time efficiency and tailored coordination structures that align with the specific disaster response context. The value of coordination varies depending on the urgency of the situation, and our results suggest that a one-size-fits-all approach to coordination is insufficient.

Although it is intuitive that bureaucratic processes discourage HOs from coordinating, our results highlight a more nuanced issue: *coalitions that are too large can be detrimental*, especially in high-urgency situations or when coordination processes are overly bureaucratic. In such cases, individual HOs may underestimate the overall time burden of coordination, resulting in efforts that ultimately hinder the relief system performance by delaying aid delivery. This finding challenges the prevailing assumption that maximum coordination is always beneficial. Instead, our results show that reducing bureaucracy is essential before striving for broader coordination, particularly to attract smaller HOs that may be understandably hesitant to engage in slow, bureaucratic processes. Excessive bureaucracy can lead to coalitions that exclude local HOs, undermining the very purpose of pooled coordination, which aims to bring together diverse actors. These challenges are exacerbated by the inherent complexities of coordinating multiple independent entities, each with its own mission, donor constraints, audits, and regulatory obligations, as well as confidentiality and security concerns in high-risk environments.

Our results also demonstrate that a partitioned model, which separates local and international HOs into sub-coalitions, can attract more HOs to participate in coordination efforts that improve overall system performance. This finding supports ongoing initiatives that advocate for more localized responses and structural adaptations that address the disparities between local and international actors^20^. These disparities, including language, cultural, geographical, and technological barriers, can be mitigated through a well-designed partitioned coordination model. For instance, coordination meetings are often conducted in English or French, using sector-specific terminology that can be difficult for local HOs to follow^20–22^. Local HOs may also lack the staffing capacity to attend frequent meetings or process large volumes of information shared by other actors^20^. In many cases, meetings are held in distant capital cities, creating logistical and financial challenges for local HOs^23^. Furthermore, reliance on internet-based platforms can hinder participation by local HOs with limited technological infrastructure^20,24^. A partitioned model can mitigate these barriers by allowing sub-coalitions to internally aggregate, translate, and communicate information, streamlining coordination and reducing the burden on smaller actors. Such a model proves particularly effective in urgent response scenarios, as it more closely aligns HOs’ decisions with the social optimal solution while minimizing the risk of over-participation.

These insights align with practical observations, where the reluctance of local HOs to participate in coordination efforts can be justified and, in some cases, beneficial to overall relief performance. For example, during the 2010 Haiti earthquake response which involved thousands of organizations^12^, some local HOs were already delivering aid in the field, while larger international HOs were delayed by lengthy coordination processes^22^. Our results suggest that the advantages of faster response times can sometimes outweigh the drawbacks of lower coordination levels, such as potential duplication of efforts. Furthermore, while the UN clusters often adopt a pooled structure, our findings support the adoption of partitioned models, similar to the South Sudan NGO Forum, particularly in urgent response contexts. However, it is crucial to note that such models cannot be designed and implemented effectively under the time pressures of disaster response. These processes should be undertaken during the preparedness phase, before a disaster strikes. Examples include the establishment of national and regional coordination platforms where local actors can exchange information, build relationships, and proactively plan for coordinated responses.

The contributions of this paper are twofold. First, although the literature typically explores the drivers and barriers of coordination through conceptual frameworks, case studies, surveys, and data analyses^11,13,25^, it often overlooks the trade-offs between these drivers and impediments. As a result, the assumption persists that more coordination invariably leads to better outcomes. This paper addresses this gap by analyzing the conditions under which higher levels of coordination may be counterproductive, particularly when urgency and bureaucracy negatively impact relief system performance. Second, most analytical studies on coordination between HOs focus on specific aspects, such as inventory exchange^26^ or resource utilization^3^, limiting our understanding of coordination at a broader systems level. We aim to fill this gap by adopting a holistic approach that incorporates multiple factors, including resource utilization, funding incentives, urgency, and bureaucracy. Lastly, prior studies tend to concentrate on a single coordination model. We compare pooled and partitioned models, demonstrating how the structure of coordination critically influences the participation of local HOs. Our findings suggest that a partitioned model can enhance local HO involvement and improve relief system performance, especially in high-urgency situations.

## Discussion and recommendations

**Fig. 2:**
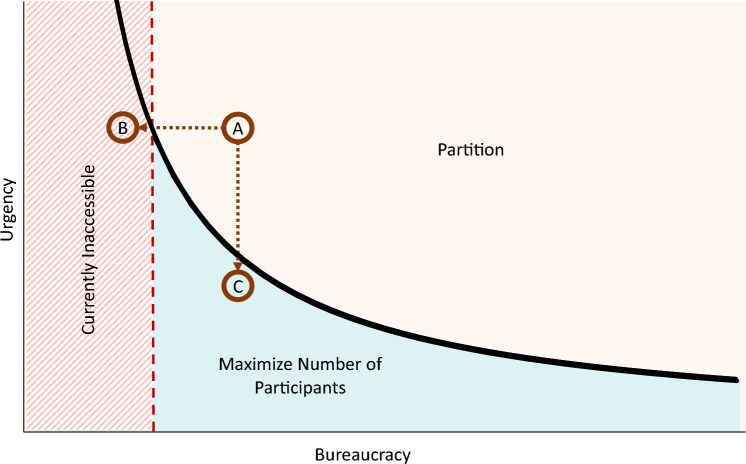
A high-level coordination model policy recommendation.

In this section, we discuss the implications of our results for practice and provide a set of recommendations to improve coordination in the sector. Figure 2 summarizes our recommendations for designing effective coordination models. In highly urgent situations such as the immediate response phase within the first week of a large-scale disaster, it may simply be infeasible to reduce bureaucratic hurdles to a level that would make maximum coordination optimal. Although harmonized efforts remain essential to effective disaster response, our findings show that its implementation, particularly when entangled with bureaucratic processes, can impose time costs that are especially problematic in urgent settings. Some degree of bureaucracy is inherent in any coordination practice, but in contexts where lives are at immediate risk, even moderate delays can undermine system performance. Accordingly, decisions about coordination should carefully weigh its time burden against the operational realities of the situation. In such cases, implementing a partitioned model can enhance coordination and improve performance, while recognizing that some duplication by uncoordinated actors is inevitable in urgent situations. The 2023 earthquakes in Türkiye provide a relevant example. Early reports of a “failed humanitarian response” highlighted delays in delivering aid to remote areas, largely attributing them to overly centralized decision-making, though limited access to regions outside government control also played a role^27^. Figure 2, point A represents this scenario, where excessive bureaucracy hampers performance. Reducing bureaucratic burdens and increasing efficiency, represented by moving towards point B, would improve coordination and overall system performance. However, achieving a fully efficient pooled structure to coordinate all HOs within the critical first 72 hours of a disaster, spread across more than 135,000 square kilometers, is virtually impossible.

At lower levels of urgency, partitioned coordination models can still enhance performance, but even modest improvements in efficiency can make coordination attractive to all HOs. Point C, for instance, represents longer-term operations after the first few weeks of response, when the acute phase has subsided. In this context, the same level of bureaucracy may allow for optimal coordination among all HOs.

### Define indicators and processes

The importance of time efficiency in coordination is often overlooked. For instance, in the 2016 Syria Humanitarian Response Plan, the UN Logistics Cluster used the number of coordination meetings held as one of its two performance indicators^28^. However, these meetings can become a significant time burden for HOs, particularly when no concrete decisions are made, or when many agenda items do not directly relate to their operations^6,20^. Our findings suggest that this not only hampers relief system performance but also discourages participation. Thus, simply counting the number of meetings is an inadequate measure of coordination success, and the sector requires more meaningful performance indicators. For example, it is crucial to evaluate whether meetings have clear, pre-communicated agendas. Similarly, the number of actionable decisions made in these meetings would serve as a far more informative indicator. Additionally, our results emphasize the need for transparency in coordination processes. Relief system performance suffers when HOs either underestimate or overestimate the efficiency of coordination. This often occurs in hastily formed and poorly structured coalitions where the time burden of coordination is uncertain. Therefore, establishing clear “rules of the game” is essential, enabling HOs to accurately assess the time demands of coordination, a critical factor in their decision-making.

### Preparedness

Designing an efficient coordination model requires significant time and effort. A key implication of our findings is that this process should not be undertaken during the response phase. A practical example is the Logistics Emergency Team, which includes four global logistics and transportation companies that collaborate with the World Food Programme to support the logistics cluster during large-scale disasters^29^. Over time, this initiative shifted its focus towards preparation, recognizing that effective field coordination is feasible and sustainable only when operational procedures are clearly defined and organizations are trained to implement them efficiently. This preparation allows them to hit the ground running when disaster strikes.

In a partitioned model, the sub-coalition of local HOs (within a country or region) can be established well in advance of a disaster. As highlighted in our field study and reported in previous research^30^, these local HOs are often already interconnected through personal relationships between their executives. Formalizing these connections by creating a network is not only feasible but can also significantly improve coordination efficiency. Such networks enable HOs to become familiar with information-sharing mechanisms and to understand each other’s capabilities, goals, and priorities. Furthermore, local HOs can participate in joint training and simulation exercises within the coordination network, enhancing preparedness.

International HOs must recognize that their local counterparts operate under different structures, cultures, and capacities. It is unrealistic to expect local actors to integrate into coordination systems designed primarily for large international HOs, which cater to their specific needs–a common concern expressed by local HOs^31^. In a partitioned model, international HOs can join the response phase by connecting with an already functioning local network. This approach aligns with the sector’s ongoing commitment to localization, as exemplified by the Grand Bargain initiative, which seeks to elevate the role of smaller, local HOs. Although implementing localization has proven challenging^32^, our findings illustrate how these efforts can strengthen relief systems by investing in local capabilities and empowering local HOs.

### Tools and platforms

Coordination efficiency hinges on the tools available for communication and information exchange. It is crucial to develop simple, standardized tools that even HOs with limited technological capacities can easily use. Standardized tools are especially valuable in time-sensitive situations, as they enable HOs to use a consistent system across multiple coordination efforts. This reduces the need for retraining and facilitates faster adoption. Effective information exchange systems can reduce the need for frequent coordination meetings, allowing HOs to focus on the data most relevant to their operations and align with other actors. The Logistics Cluster Information Exchange (LogIE) platform is one example, providing a user-friendly interface for sharing and accessing pre-processed information^33^. However, it is essential to distinguish between information exchange and coordinated action. The latter requires clear agreements and commitment. Time efficiency is critical, and HOs should not be forced to navigate through excessive information to make coordinated decisions. This underscores the vital role of the Emergency Response Coordinator and highlights both the potential and limitations of information-sharing platforms.

## Method

Game theory provides a robust framework for analyzing the interconnected decisions of multiple actors^18^. It is therefore a suitable method in our settings as HOs’ coordination decisions affect not only their own performance but also that of other HOs and the overall relief operations. Accordingly, we develop a game-theoretical model to explore HOs’ coordination choices, considering both their individual perspectives and the objective of the central coordinator who aims to optimize system performance.

Game-theoretical models are generally divided into cooperative and non-cooperative games. In a cooperative game, players make “binding commitments regarding which strategy they will choose before they actually choose their strategies”^18^. Non-cooperative games can also involve agreements between players, but these are made freely according to what each player considers best for themselves^34,35^. In humanitarian settings, no single organization holds authority over others. This means that the central coordinator cannot enforce coordination through the exertion of power. HOs retain autonomy as they have different missions and are bound by different donor restrictions and regulations. Therefore, similar to other studies in humanitarian settings^26^, we develop our model as a non-cooperative game to better reflect the real world.

## Model settings and assumptions

We focus on the aftermath of a large-scale disaster, where both international and local HOs are involved in relief efforts, heightening the complexity and importance of coordination. At the heart of the problem is the formation of a *coalition*, defined as the group of HOs that actively participate in coordination. Effective coordination requires not just information sharing but genuine commitment and resource investment from all actors^36^. Thus, we define *participation* as an HO’s active role in preparing, sharing, and analyzing information (e.g., needs assessments, accessibility mapping) and ensuring their actions are aligned with others (e.g., task division, resource allocation). The model assumptions are outlined below.

### Capacity

In line with real-world practices, we assume that each HO (i.e., player) has a predetermined capacity for the relief operations. For example, the International Federation of Red Cross and Red Crescent Societies (IFRC) sets early targets for the number of people they intend to assist and the quantity of relief items to distribute within the first two weeks, based on available funding and the disaster’s scale. To build a high-level model, we define an HO’s capacity as its total ability to meet demand during the short-term post-disaster period. This encompasses activities such as shipping inventory to the field, financial resources for procurement, and the human resources needed for logistics. Additionally, as we focus on large-scale disasters, we assume that total demand exceeds the combined capacities of all HOs.

### Player types

Given the importance of HO heterogeneity and the critical role of local HOs in relief operations^13,32^, we categorize HOs into two types: local and international. For simplicity, we assume homogeneity within each type. However, the model and findings can be extended to more general cases that account for a broader range of HOs with more detailed categorizations. (See e-companion A for a general model with heterogeneous HOs.) We assume that international HOs have larger capacities than local HOs, an assumption supported by our field study. In the context of our model, which focuses on large-scale disasters, international HOs typically become involved when local capacity is insufficient.

### Decision-making

In large-scale relief operations, HOs typically join coordination efforts in a sequential manner. Local HOs often begin their relief efforts immediately, operating independently and without coordination. In contrast, international HOs face delays in obtaining authorization, traveling, and setting up operations. For instance, after the Türkiye-Syria earthquakes, local HOs rapidly initiated emergency operations, but reported being unaware of the arrival or plans of international HOs that began arriving later^37^. These delays allow international HOs time to establish early communication and coordination before their field operations start. Humanitarian practitioners in our field study stressed that international HOs frequently have pre-existing relationships, either from development programs or previous response efforts, as well as personal connections among executives. As a result, large-scale relief coalitions are often driven by large international HOs^20^. Clusters must actively reach out to local HOs and invite them to join the coalition^24^. Our model reflects this sequential process, considering two possible sequences where either local HOs or international HOs make decisions first. We also account for scenarios where players are either *myopic* or *forward-looking*: a myopic player considers only the decisions of preceding players, while a forward-looking player anticipates the decisions of subsequent players. Myopic decision-making is relevant in situations where HOs lack information about the future participation of others in coordinated efforts. For example, during the 2023 earthquakes in Türkiye, some local HOs reported being unaware of international actors’ plans to respond – “they often did not know they were arriving until they were on site.”^37^ In line with non-cooperative game theory^18^, we assume that each player seeks to maximize their own utility. This mirrors the competitive nature of humanitarian contexts involving diverse organizations that are competing over limited funds and media attention^15^.

### Utility function

At its core, an HO’s utility combines the services it provides and its income^15^. In the context of this paper, service translates to minimizing deprivation of the people in need^38^ by maximizing the number of affected people served (*demand coverage*) within an acceptable response time (*timeliness*). We consider HOs’ two main income sources: *donations* from the public and *grants* from governments and institutions. Incorporating all of these elements is crucial for developing a realistic model. This enables the model to capture both improved service provision and the competition that exists between HOs.

#### Timeliness

Time pressure is a critical factor in relief operations, particularly following large-scale disasters^14^. Urgency often leads to bypassing strategic decision-making at the operational level^11^, and delayed aid delivery results in increased deprivation costs for affected populations. We define urgency based on the immediacy and severity of risks to human life and well-being. Disasters that place large numbers of people in life-threatening conditions with limited time for intervention (e.g., earthquakes and floods) exhibit higher urgency than those where needs are significant but not immediately life-threatening (e.g., displacement without direct threats to survival). Likewise, delivering life-saving aid is more urgent than providing infrastructural support or non-critical services. Nevertheless, in disaster response, a wide range of relief items and services are urgently needed to ensure survival and basic well-being. Accordingly, our model is not restricted to any specific type of service.

To represent how delays interact with urgency, we draw on research that suggests human suffering from deprivation over time is best modeled using nonlinear increasing functions^38,39^. Notably, logistic growth and convex forms are nearly identical shortly after disasters^39^. Reflecting this dynamic, and the potentially severe consequences of delayed response in high-urgency settings, we penalize response delays using a convex exponential function.

Coordination can further delay response operations due to bureaucratic processes^40,41^. For example, bureaucratic hurdles slowed resource mobilization during the Sierra Leone Ebola response^6^. As more players join, the time required to analyze shared information and make collective decisions increases^42^. However, as commonly cited by practitioners, the relationship between coalition size and response delays is not linear but rather concave, as coalitions tend to adapt their decision-making processes as they grow^20^. We thus model the additional time due to bureaucracy as concave in relation to the number of players, assuming a natural logarithmic form. This captures HOs’ concerns about bureaucratic delays without imposing excessive penalties for larger coalitions. It also enables closed-form analytical results. To ensure our findings are not driven by this assumption, we performed extensive numerical simulations using alternative concave forms, which confirms the robustness of our main insights (see e-companion D).

#### Demand coverage

HOs aim to maximize demand coverage during disaster response through coordination^3^. In our field study, practitioners noted that without coordination, HOs often “target the same families with the same item, resulting in duplication and wasted resources.” This lack of coordination, as seen in the 2017 Rohingya refugee crisis response, reduces overall demand coverage^43^. We assume that resource utilization benefits increase linearly with coalition size, as outlined below.

Coordination improves resource utilization in two key ways. First, as members are informed of each other’s activities and align their operations, duplication of efforts is minimized. For example, consider *n* HOs providing relief to $R \ge n$ regions^3^. Each HO selects a region to operate in, with a probability of duplication if another HO also selects that region. Without coordination, the probability of duplication is $\nicefrac {(n-1)}{R}$. However, if an HO is aware of the selections of *i* other HOs, the probability of duplication decreases to $\nicefrac {(n-1-i)}{R}$, demonstrating a linear relationship in *i*.

Second, HOs gain valuable insights into field conditions, demand, and available supplies from other members^13^. Information scarcity is a major challenge in large-scale disasters. Although the value of insights may diminish as more members join the coalition (due to limited supplies or overlapping assessments)^44^, we assume a linear relationship for simplicity. Our analysis acknowledges that this may overestimate the benefits of coordination, but it allows us to explore the assumption that more coordination always leads to better outcomes, ensuring that the model does not prematurely dismiss the potential benefits of higher levels of coordination.

#### Donations

Research indicates that HOs’ donation income is influenced by their reputation^45^, which is closely tied to media exposure^15^. Competition for media attention has been identified as a barrier to coordination^15^. For example, reports linked this competition to failures in the Haiti response^16^. This dynamic often incentivizes HOs to operate independently to claim achievements and attract media attention. On the other hand, participation in a coalition can enhance an HO’s visibility and reduce the risk of being perceived as uncooperative. One HO executive noted that despite the challenges, *“HOs may still decide to coordinate to gain media visibility and avoid negative publicity.”*

These opposing incentives highlight the complex role of media exposure. To address this, we develop a holistic model that accounts for the effect of coordination on media attention—and consequently donations—ranging from fully competitive (negative) to fully synergistic (positive). In competitive scenarios, joining a larger coalition dilutes media attention and reduces donation income. Conversely, in synergistic scenarios, coalition growth amplifies media visibility. Given the lack of strong evidence for nonlinearity in this context, we assume a linear relationship for simplicity and tractability.

#### Grants

Assuming coordination enhances performance, large donors encourage HOs to work with their peers, and participating in a coalition is considered an advantage to win grants^46^. Some donors require joint appeals for fund allocation, e.g., Central Emergency Response Funds. Therefore, joining a coalition can unlock access to funds unavailable to HOs working alone. The main benefits, however, are enabled by HOs’ improved performance in the field when they coordinate their efforts. Unlike public donors who are limited in their ability to assess HOs’ operations, institutional donors can inspect HOs more closely and grant funds based on operational performance^15^. Therefore, we assume that an HO’s chances to receive grants from large donors increase with its demand coverage. Similar to demand coverage and donations, we assume a linear relationship as the literature does not provide strong evidence for nonlinearity.

### Relief system performance

The central coordinator, e.g., the Emergency Response Coordinator who chairs the UN Inter-Agency Standing Committee^47^ works closely with the country’s authority to obtain the social optimal solution, i.e. the best relief system performance. Not only individual HOs, but also society benefits from higher demand coverage, better timeliness of response, and HOs’ sustainability to continue providing aid in the future. We therefore define relief system performance as the sum of the utilities of all HOs involved in the operations, aligned with non-cooperative game models evaluating system optimal solutions^18^. This guarantees that any disparities observed between HOs’ decisions and the social optimal solution stem from the fact that HOs focus on their own utility rather than the collective effects on the system. In essence, this safeguards against misalignments arising from different evaluation criteria.

### Partitioning

Partitioning enhances coordination efficiency by addressing key barriers between local and international HOs. For instance, during the UN clusters’ attempts at inclusivity in Pakistan, local HOs struggled to participate effectively due to resource constraints and the volume of information shared by other actors^20^. Language barriers can also lead to time lost in translation and interpretation^20–22^. Partitioned sub-coalitions can mitigate these challenges by internally aggregating and adapting information to members’ needs. They also reduce the cognitive and logistical burden on smaller actors and prevent redundancies in discussions and decisions specific to each type of HOs. Furthermore, partitioning allows HOs to more clearly convey their knowledge and needs, reducing the risk of misinterpretations and translation errors. Local HOs can coordinate more effectively within a culturally aligned sub-coalition, using their native language and sharing a contextual understanding of the region. However, partitioning may reduce some benefits of coordination, such as the loss of information specific to each sub-coalition. Therefore, effective coordination between sub-coalitions is critical to maximizing the advantages of partitioning. In our model, partitioning is effective because key information is easily shared between sub-coalitions via a central coordinator like the Emergency Response Coordinator. This preserves the benefits of coordination while reducing the impact of the number of participants in one sub-coalition on the response time of the other.

## Pooled model

Consider the problem with two types of players: local and international HOs. $n_1$ players of type 1 and $n_2$ players of type 2 decide whether to join a coalition with all participants pooled into one group. Players of type 1 make their decisions first and $y_1$ HOs join the coalition. Then, type 2 HOs decide and $y_2$ of them join. A central coordinator, on the other hand, decides on the optimal number of HOs of each type to be in the coalition to maximize the relief system performance. Notations are presented in Table 1.

**Table 1:** Notations.

Indices and variables	
*k*	Player type index, $k \in \{1,2\}$
$y_k$	Number of type *k* HOs in the coalition ($0\le y_k \le n_k$)
$y_k^o$	Number of type *k* HOs in the coalition in the social optimal solution
$y^o$	Social optimal coalition size
$y_k^*$	Number of type *k* HOs in the coalition in the equilibrium solution
$y^*$	Equilibrium coalition size
Parameters	
$\alpha _k$	Marginal benefits of coordination for players of type *k*
$\alpha _{Ck}$	Relative importance of demand coverage in utility of type *k* HOs
$\alpha _{Dk}$	Relative importance of donations in utility of type *k* HOs
$\alpha _{Gk}$	Relative importance of grants in utility of type *k* HOs
$\alpha _{T}$	Relative importance of deprivation cost due to timeliness of response in HOs’ utility
$\beta$	Time burden of coordination
$\gamma$	Increase in resource utilization per coalition member ($0 \le \gamma n \le 1$)
$\eta$	Partitioning level ($0<\eta =1-\rho \le 1$)
$\theta$	Media competition level ($-1 \le \theta \le 1$)
*b*	Exponential deprivation parameter
$c_{k}$	Capacity of a type *k* HO for its efforts during relief operations (*T*)
*M*	Media coverage level of the response
*n*	Number of HOs active in the response
*T*	Baseline response time
$t_{B}$	Unit bureaucracy time increase by joining the coalition
$u_0$	Relief system performance if HOs do not coordinate
$w_{Ci}$	Weight of demand coverage in HO *i*’s utility function
$w_{Di}$	Weight of public donation income in HO *i*’s utility function
$w_{Gi}$	Weight of grants income in HO *i*’s utility function
Functions	
$U_k(y)$	Change in utility of a type *k* HO by joining a pooled coalition of size *y*
$\hat{U}(y_1,y_2)$	Relief system performance when $y_1$ HOs of type 1 and $y_2$ HOs of type 2 join a pooled coalition
$U_Dk(y_1,y_2)$	Change in utility of a type *k* HO by joining a partitioned coalition of $y_1$ type 1 and $y_2$ type 2 HOs
$\hat{U}_D(y_1,y_2)$	Relief system performance when $y_1$ HOs of type 1 and $y_2$ HOs of type 2 join a partitioned coalition

### Utility function

**Fig. 3:**
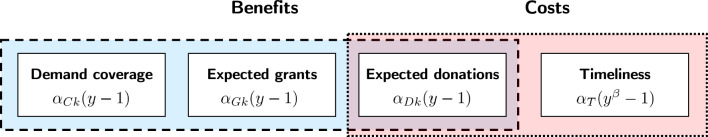
Components of the utility function $U_{k}(y)$.

The utility function is formulated based on the assumptions presented above. It is the total of the four utility components illustrated in Fig. 3.

Coalition size increases response time logarithmically. We express the total response time for a player in a coalition of *y* participants as $T + t_B \ln (y)$, where $t_B$ is the incremental bureaucracy time from joining the coalition, and *T* represents the baseline response time when an HO operates alone during the short-term post-disaster relief period. Timeliness of response is then translated into disutility in the HO’s utility function. Using an exponential function with parameter *b*, the increase in timeliness disutility from joining a coalition of size *y* is given by $e^{b(T + t_B \ln (y))}-e^{bT} = e^{bT} ( y^{bt_B}-1)$.

Joining a coalition increases an HO’s utility through improved demand coverage and grant income. Depending on the level of competition for media attention, it either increases or decreases the donations the HO receives. We assume these changes are proportional to the coalition size. Thus, the overall change in utility for an HO of type *k* from joining a coalition of size *y*, relative to not joining, is given by

1 $$\begin{aligned}&U_{k}(y)= \alpha _k (y-1) - y ^ \beta + 1 ,&\forall k=1,2 \end{aligned}$$

where $\beta =b t_B$ represents the time burden of coordination and $\alpha _k=\displaystyle \frac{\alpha _{Ck}+\alpha _{Gk}+\alpha _{Dk}}{\alpha _{T}}>0$ is the marginal benefit of coordination for players of type *k*. We describe these elements below.

The relative importance of deprivation cost due to timeliness of response is captured by $\alpha _{T}=e^{bT}$. Thus, urgency of response, *b*, increases the weight of deprivation cost in HOs’ utility functions. Each type of HO assigns a weight to demand coverage in its utility function, denoted by $w_{Ck}$. The HO’s capacity, $c_k$, allows it to cover more demand. Additionally, greater operational overlap between HOs, and thus a higher likelihood of duplication, makes demand coverage more critical in coordination decisions. This is represented by $\gamma$, the increase in resource utilization per coalition participant, where $\gamma n < 1$. Therefore, the relative importance of demand coverage in the utility of a type *k* HO is given by $\alpha _{Ck} = w_{Ck} \gamma c_k$.

In modeling the role of income incentives, we consider that each type of HO puts different weights on income from grants vs. donations. For a type *k* HO, these weights are represented by $w_{Gk}$ and $w_{Dk}$, respectively. As grants are sensitive to performance, higher dependence on grants makes resource utilization, $\gamma c_k$, more important in an HO’s utility function. Thus, the relative importance of grants in the utility function of a type *k* HO equals $\alpha _{Gk}=w_{Gk}\gamma c_k$. Public donations depend on the total media coverage the disaster receives, denoted by *M*, and media competition level, $\theta$. We have $-1 \le \theta \le 1$, reflecting the negative or positive reaction to coordination. Thus, the relative importance of public donations in a type *K* HO equals $\alpha _{Dk}=w_{Dk} M \theta c_k$. (We present the comparative statics of the utility function in e-companion B.)

### Equilibrium

We first take the HOs’ perspective to determine the equilibrium. When decision-making is myopic, type 1 HOs make their decisions first. An HO joins a coalition of $y_1-1$ other HOs only if $U_1(y_1) \ge 0$. Therefore, from (1), a total of $y^*_1$ type 1 HOs join the coalition such that

$$\begin{aligned} y_1^*=\max \{y:\alpha _1 y - y^\beta -\alpha _1 +1 \ge 0\}. \end{aligned}$$

Type 2 HOs are next in the sequence and make their decisions knowing that $y^*_1$ HOs are in the coalition. Therefore, a number equal to $y^*_2$ join, making an equilibrium coalition of size $y^*=y^*_1+y^*_2$ such that

$$\begin{aligned} y^*_2=y_2^*(y_1^*)=&\max \{y_2:\alpha _2 (y^*_1+y_2) - (y^*_1+y_2)^\beta -\alpha _2 +1 \ge 0\}. \end{aligned}$$

When players are forward-looking, $y^*=y_1^*(y_2^*)+y_2^*(y_1^*)$ players will form a coalition such that

$$\begin{aligned} y_1^*(y_2^*)=&\max \{y_1:\alpha _1 (y_1+y^*_2) - (y_1+y^*_2)^\beta -\alpha _1 +1 \ge 0\}\\ y_2^*(y_1^*)=&\max \{y_2:\alpha _2 (y^*_1+y_2) - (y^*_1+y_2)^\beta -\alpha _2 +1 \ge 0\}. \end{aligned}$$

### Social optimal

The central coordinator seeks to determine the number of HOs of type 1 ($y_1$) and type 2 ($y_2$) to maximize relief system performance, $\hat{U}(y_1,y_2)$ defined in Equation (2). $u_0$ is the performance if HOs do not coordinate (i.e., $y_1=y_2 = 0$).

2 $$\begin{aligned}&\hat{U}(y_1,y_2)= u_0 + y_1 U_1(y_1+y_2) + y_2 U_2(y_1+y_2) \end{aligned}$$

The central coordinator seeks to solve the following problem over integer values of $y_k$ where $y_k \le n_k$, $\forall k=1,2$.

$$\begin{aligned} \max _{y_1,y_2 \ge 1}\hat{U}(y_1,y_2). \end{aligned}$$

Thus, the social optimal coalition includes a total of $y^o=y^o_1+y^o_2$ HOs, i.e., $y^o_1$ HOs of type 1 and $y^o_2$ HOs of type 2 where

$$\begin{aligned} \left( y^o_1(y^o_2),y^o_2(y^o_1)\right) =\mathop {\mathrm {arg\,max}}\limits _{y_1,y_2 \ge 1} \hat{U}(y_1,y_2). \end{aligned}$$

## Partitioned model

We now investigate the partitioned model where local and international HOs form separate sub-coalitions that are coordinated at the level of the central coordinator. Let us denote the level of partitioning between the sub-coalitions by $\eta$, where $\eta \in [0,\eta _0)$ and $\eta _0<1$. When $\eta =0$, all participants directly coordinate with each other, making the model equivalent to the pooled model. As $\eta$ increases, the sub-coalitions are more separated. Therefore, the number of players of each type has a smaller impact on the response time of players of the other type. $\eta _0$ is the maximum level of partitioning at which the central coordinator can ensure an efficient coordination between the sub-coalitions such that the same benefits are achieved. For instance, the central coordinator can split the geographical response area into two subsets of regions, assigning each to one sub-coalition. The two smaller groups can then coordinate their response within their designated areas in parallel. Some higher-level coordination between the sub-coalitions remains necessary. Yet, this approach allows for quicker decision-making while avoiding duplication between the two groups.

The change in the utility of a type *k* HO from joining the coalition consisting of a sub-coalition of $y_1$ players of type 1 and a sub-coalition of $y_2$ players of type 2 is given in Equation (3). Equation (4) calculates the resulting relief system performance.

3 $$\begin{aligned} U_{Dki}(y_1,y_2) =&\alpha _k (y_k+y_{3-k}-1) - [(y_k+ (1-\eta ) y_{3-k}) ^ \beta - 1],&\forall k=1,2 \end{aligned}$$

4 $$\begin{aligned} \hat{U}_{D}(y_1,y_2) =&u_0 + \alpha _1 y_1^2 + \alpha _2 y_2^2 + (\alpha _1+\alpha _2) y_1 y_2 - (\alpha _1 y_1 + \alpha _2 y_2) + y_1 + y_2&\nonumber \\&- y_1(y_1+(1-\eta ) y_2)^\beta - y_2(y_2+(1-\eta ) y_1)^\beta&\end{aligned}$$

## Analysis and propositions

**Table 2:** Summary of the results.

	**Low time burden**		**High time burden**	
	**Myopic**	**Forward-looking**	**Myopic**	**Forward-looking**
Equilibrium coalition size is equal to or	Smaller than optimal		Larger than optimal	
By partitioning, alignment between the equilibrium and the social optimal	Decreases		Increases	
Partitioning leads to the same or higher	Number of participants, optimal coalition size, optimal system performance			
	Equilibrium system performance			

This section presents our analysis results and propositions, summarized in Table 2. We first evaluate when participation of more HOs in coordination improves relief system performance by comparing the number of HOs that join a coalition with the optimal number that maximizes system performance. Next, we investigate the impact of partitioning by comparing the pooled and partitioned models in terms of number of participants, system performance, and alignment between the equilibrium and social optimal solutions. Proofs are presented in e-companion C.

### Proposition 1a

When $\beta <1$, the social optimal coalition is at least as large as the equilibrium coalition, i.e., $y^o \ge y^*$.

### Proposition 1b

When $\beta>1$, the equilibrium coalition is at least as large as the social optimal coalition, i.e., $y^* \ge y^o$.

Proposition 1a is in line with the assumption that HOs should aim for maximum coordination and that they need to be encouraged to increase their coordination efforts. However, this holds only when urgency is low and/or coordination processes are efficient and not very bureaucratic. In such scenarios (i.e., when $\beta <1$), small HO participation might fall short of the optimal level as they ignore the positive externalities of their involvement. We note that this outcome is not necessarily due to myopic decision-making. Small HOs may refrain from joining the coalition to operate faster, despite the potential benefits for large HOs and overall system performance.

On the other hand, Proposition 1b indicates the risk of coalitions that are too large when urgency and/or bureaucratic burdens are high. This situation ($\beta>1$) leads to a convex increasing deprivation function. Therefore, one HO’s decision to join the coalition not only increases its own timeliness disutility, but also those of all other participants. This is a negative externality that HOs ignore. Regardless of being myopic or forward-looking, each HO only focuses on its own utility, neglecting the overall increase in response time for all participants. This negligence can lead to a worse relief system performance and cause HOs to form a larger coalition than necessary.

We also find that when time burden of coordination is low, small HOs are more inclined to join a coalition where large HOs are present, thereby enabling the advantages of pooling both types of HOs in a single coalition. Conversely, under high time burden of coordination, a pooled model can lead to exclusion of small HOs. This absence stems from anticipating significant delays resulting from the participation of multiple large HOs (see e-companion B).

### Proposition 2a

As the level of partitioning ($\eta$) increases, the number of coordinating HOs increases or remains the same. When time burden is high or HOs are forward-looking, this leads to a better or identical relief system performance.

### Proposition 2b

As the level of partitioning ($\eta$) increases, the social optimal coalition size and the optimal relief system performance increase or remain the same.

Partitioning allows HOs to spend less time coordinating their efforts with many HOs of a different type, allowing more HOs to join and benefit from coordination. This especially encourages active participation of local HOs. When time burden is high or HOs are forward-looking, the increase in the number of participating HOs leads to a better relief system performance as well. If time burden is low and HOs are myopic, it is possible that local HOs join a partitioned coalition while their inclusion eventually lowers relief system performance. We tested the occurrence of this phenomenon by numerical experiments presented in e-companion D. In our experiments, we observe that this occurs only on rare occasions at relatively higher levels of coordination time burden.

A social optimal partitioned coalition includes more HOs than the pooled model social optimal solution, owing to enhanced coordination efficiency between different types of HOs. Regardless of whether coordination time burden is high or low, an effective partitioning not only facilitates increased coordination but also enhances overall system performance.

### Proposition 3

When $\beta <1$ ($\beta>1$), as the level of partitioning ($\eta$) increases, the difference between the equilibrium solution and the social optimal increases (decreases) or remains the same.

This result shows that a central coordinator (and thus society) observes more benefits in partitioning than individual HOs. A key finding in the pooled model was the possibility of equilibrium coalitions that are larger than social optimal when coordination time burden is high ($\beta>1$). We find that partitioning can alleviate this problem and increase the alignment between HOs’ decisions and the social optimal solution. It makes HOs willing to join larger coalitions and increases the social optimal coalition size. Importantly, the latter grows more than the equilibrium coalition size, leading to a coalition that is closer to the optimal.

On the other hand, when time burden is low ($\beta <1$), small HOs may still underestimate the collective benefits for all participants. In these situations, an optimal partitioned coalition includes small HOs that are not included in the optimal pooled coalition. However, if marginal benefits of coordination for small HOs are lower than a threshold, they still choose to operate alone. Therefore, when the urgency of the relief operations subsides, a partitioned system has more to gain from small HO participation than a pooled system.

## Limitations and future research

In this paper, we developed a high-level model to improve our understanding of coordination between HOs, considering relief context and coordination models. This approach has limitations that offer opportunities for future research.

First, motivated by a range of practice-related factors, including persistent critiques about the limited inclusion of local HOs in coordination efforts, inefficiencies in coordination between international and local HOs, and the potential to establish networks of local HOs during the preparedness phase, our analysis focused on coordination among international and local non-governmental HOs. In practice, however, disaster response involves a broad spectrum of organizations that can be categorized along various dimensions, such as functional specialization (e.g., health or shelter, as reflected in the cluster system) and geographic orientation (i.e., target population or area). Although our model and findings can be extended to incorporate more nuanced categorizations of HO heterogeneity (see e-companion A), future research could explore these complexities in greater depth. This includes examining nested levels of coordination, such as task-specific Technical Working Groups within local sub-coalitions and comparing different partitioning logics.

Second, our insights are derived from an analytical model informed by the literature and a field study that also helped validate the relevance of our findings. We further tested the robustness of our results through extensive numerical simulations. Future research can complement this work by leveraging detailed empirical data to expand and refine our understanding of coordination dynamics in diverse operational contexts.

Third, we define coordination as joint decision-making aimed at aligning HOs’ operations. This scope aligns with most prevalent coordination mechanisms such as the UN clusters, that primarily function at the alignment level^20^, and excludes resource sharing which involves additional challenges and different dynamics. Given the potential value of resource sharing, particularly for smaller HOs that can benefit from the capacities of larger international partners, future research could explore collaborative models and investigate mechanisms such as the stock exchange proposed in some prior studies^26^.

## Supplementary Information

Below is the link to the electronic supplementary material.

Supplementary material 1

## Data Availability

The datasets generated and analysed in this paper are available from the corresponding author upon request.
