# Supplementary material for: Analyzing coordination structures for effective humanitarian relief operations
Source: Sci Rep. 2026 Jan 11;16:1327. doi: 10.1038/s41598-025-33588-1 (PMC12796352; doi:10.1038/s41598-025-33588-1)
Supplement: Supplementary file 1 — Supplementary material 1 [file 41598_2025_33588_MOESM1_ESM.pdf]

E-Companion for Analyzing Coordination Structures for Effective Humanitarian Relief Operations

Iman Parsa
Department of Entrepreneurship, Innovation and Technology, Stockholm School of Economics, Stockholm, Sweden.
iman.parsa@hhs.se

Mahyar Eftekhar, Scott Webster
W.P. Carey School of Business, Arizona State University, Tempe, Arizona.

Luk N. Van Wassenhove
Technology and Operations Management, INSEAD, Fontainebleau, France.

A. General pooled model

The model can easily be generalized to include n heterogeneous HOs. We use index i to indicate the position of an HO in the sequence, i.e., HO i decides whether to join after players 1 to $i-1$ have made their decisions. HO i decides whether to join the coalition ($x_i = 1$), or not, ($x_i = 0$). Let $\mathbf{x}_{-i} = \{x_1, \ldots, x_{i-1}, x_{i+1}, \ldots, x_n\}$ denote the vector of coordination decisions by HOs other than i, where x_j indicates the known decision of player j when $j \leq i$ and the anticipated decision of player j when $j > i$. We define $N(\mathbf{x}_{-i})$ as the number of players in the coalition excluding player i, i.e., $N(\mathbf{x}_{-i}) = \sum_{j \neq i} x_j$.

Based on the assumptions presented in the paper, we can derive $U_i(\mathbf{x}_{-i})$, the change in HO i's utility from joining the coalition relative to not joining as follows.

$$U_i(\mathbf{x}_{-i}) = \alpha_i N(\mathbf{x}_{-i}) - [1 + N(\mathbf{x}_{-i})]^\beta + 1, \qquad \forall i = 1, \ldots, n \tag{A.1}$$

B. Comparative statics

B.1. Benefits of coordination:

Marginal benefits of coordination for HO i equals $\alpha_i = [(w_{Ci} + w_{Gi})\gamma + w_{Di} M\theta] c_i e^{-bT}$. Thus, marginal benefits are increasing in the waste factor, γ, which increases the impact of coordination on resource utilization. This increase is higher when the weights of demand coverage and income from grants (i.e., w_{Ci} and w_{Gi}) are greater. When media attention is synergistic (competitive), i.e., $\theta > 0$ ($\theta < 0$), marginal benefits of coordination are higher for disasters with greater (smaller) media coverage, M. Larger HOs owning higher capacity obtain higher marginal benefits of coordination and are more sensitive to changes in the aforementioned parameters. Finally, from (A.1), the total benefits of coordination for each HO, $\alpha_i N(\mathbf{x}_{-i})$, is linearly proportional to the number of HOs in the coalition.

B.2. Costs of coordination:

Marginal costs of coordination increase as β, the time burden of coordination, increases. This factor increases when deprivation parameter, b, is high, as is the case when urgency is high and delays in delivering the services result in high levels of deprivation (e.g., medical relief after a disease outbreak). The level of bureaucracy in coordination, t_b, is the other critical parameter.When coordination is time consuming and involves a high level of bureaucracy (e.g., when there are multiple small and large HOs with differences in language, culture, and interests), coordination time burden increases. This increase in marginal costs changes exponentially as the coalition size increases. From (A.1), we have

$$\frac{\partial U_i(\mathbf{x}_{-i})}{\partial \beta} = -\ln(1+N(\mathbf{x}_{-i}))(1+N(\mathbf{x}_{-i}))^{\beta} < 0,$$

showing that if $\beta > 1$, marginal change in the coordination time burden significantly increases HOs' coordination costs, especially in larger coalitions. However, if urgency is low or the system is time efficient (i.e., $\beta < 1$, so that the addition of new members does not incur a large time cost), coordination decisions are less sensitive to the exact value of β. In practice, during the short post-disaster period, HOs barely know the exact value of t_B, and so rely on their intuitive estimate of this factor. If they find the coordination system to be time efficient, they are more willing to take the risk of having underestimated the time cost. But, if they perceive the system to be inefficient, they are sensitive to the time cost since a small increase in the level of bureaucracy leads to a large change in their costs.

C. Proofs

Player i joins a coalition if and only if it increases its utility, i.e., if $U_i(\mathbf{x}_{-i}) > 0$. Therefore, player i joins a coalition of $y-1$ HOs if

$$U_i(\mathbf{x}_{-i}) = \alpha_i(y-1) - \Big([1+y-1]^{\beta} - 1\Big) > 0 \Rightarrow \alpha_i > \frac{y^{\beta}-1}{y-1}.$$

Proposition 1a

When decision making is myopic, players of type 1 will not form a coalition unless $U_1(2) > 0$. For $k = 1, 2$ we have

$$U_k^{'}(y) = \alpha_k - \beta y^{\beta-1}$$
$$U_k^{''}(y) = -\beta(\beta-1)y^{\beta-2}.$$

Therefore, if $U_1(2) > 0$, $U_1(y)$ is increasing convex for all $y_1 \geq 2$ and therefore $y_1^* = n_1$ is the utility-dominant equilibrium. We have $U_1(2) > 0$ if $\alpha_1 > 2^{\beta} - 1$.

Then, among players of type 2 that are next in the sequence, knowing that y_1^* players are in the coalition, a number equal to y_2^* will join such that $U_2(y_1^* + y_2^*) = 0$, if $U_2(y_1^*) > 0$, i.e., if

$$\alpha_2 > \frac{{y_1^*}^{\beta} - 1}{y_1^* - 1} \tag{C.1}$$

Therefore, when $\alpha_1 > 2^{\beta} - 1$, if we also have $\alpha_2 > \dfrac{{n_1}^{\beta} - 1}{n_1 - 1}$, then a number of $y_2^* = n_2$ players of type 2 will join the coalition. Note that $U_2^{'}(n_1 + y_2) = \alpha_2 - \beta(n_1 + y_2)^{\beta-1} > 0$ when (C.1) holds. Also, we have $\dfrac{{n_1}^{\beta} - 1}{n_1 - 1} < 1$. Therefore,

if $\dfrac{{n_1}^{\beta}-1}{n_1-1}<\alpha_2<1<\alpha_1$ a sequence where players with α_2 precede leads to a smaller coalition than the opposite sequence where players with α_1 join first. When considered first in the sequence, players with α_2 decide not to join the coalition, but they all would join if they know players of type 1 are in the coalition.

We now analyze the social optimal solution, first considering the case that only HOs of type 2 are in considered. We have

$$\hat{U}^{'}_{y_2}(0,y_2)=\frac{\partial\hat{U}(0,y_2)}{\partial y_2}=2\alpha_2 y_2-(\beta+1)y_2^{\beta}-\alpha_2+1$$
$$\hat{U}^{''}_{y_2}(0,y_2)=\frac{\partial^2\hat{U}(0,y_y)}{\partial y_2^2}=2\alpha_2-(\beta+1)\beta y_2^{\beta-1}$$

When $\beta<1$, we have $\lim_{y_2\to\infty}\hat{U}^{'}_{y_2}(0,y_2)=\infty$. Therefore, ignoring the constraint on the number of players, the social optimal coalition size equals ∞ at any value of $\alpha_2>0$.

Further, if

$$\alpha_2>\beta \tag{C.2}$$

We have $\hat{U}^{'}_{y_2}(0,1)=\alpha_2-\beta>0$, and

$$\frac{\beta+1}{2}<1<\frac{\alpha_2}{\beta} \tag{C.3}$$

Note that

$$\hat{U}^{''}_{y_2}(0,y_2)=2\beta\Big(\frac{\alpha_2}{\beta}-(\frac{\beta+1}{2})(\frac{1}{y_2})^{1-\beta}\Big).$$

Therefore $\hat{U}^{''}_{y_2}(0,1)>0$, and $\hat{U}^{''}_{y_2}(0,y_2)$ is increasing in y_2. Thus, $\hat{U}(0,y_2)$ is convex increasing for all $y_2\geq 1$ and therefore the optimal coalition includes all n_2 HOs of type 2 for any $\alpha_2>\beta$ and $n_2>1$.

If $\alpha_2<\beta$, then $\hat{U}(0,y_2)$ is initially decreasing concave at $y_2=1$ ($\hat{U}(0,1)=u_0$). As y_2 continues to increase, $\hat{U}(0,y_2)$ shifts to convex decreasing, and after reaching a local minimum, it eventually becomes increasing convex. Therefore, the social optimal coalition includes n_2 HOs of type 2 as long as $\hat{U}(0,n_2)>u_0$. For a given n_2, we have

$$\hat{U}(0,n_2)-u_0=\alpha_2 n_2^2-(\alpha_2-1)n-n^{\beta+1}>0\Rightarrow\alpha_2>\frac{n_2^{\beta}-1}{n_2-1}$$

Note that $\dfrac{n_2^{\beta}-1}{n_2-1}<\beta$ for $n_2>1$ and $\beta<1$. Also note that, since $\beta<1$, the minimum threshold for α to make a coalition of all n_2 HOs the social optimal is smaller for larger values of n_2. In other words, if $\beta<1$ and we have a large enough pool of players of the same type, the social optimal is to form a coalition including all players even when the marginal benefits of coordination are very low.

If $\alpha_2\leq\dfrac{n_2^{\beta}-1}{n_2-1}$, we have $\hat{U}(0,y_2)\leq u_0$ for all $y_2>1$. Therefore, the social optimal solution is to have no coalition.

We now consider the case where two types of HOs are considered simultaneously. Note that

$$\nabla\hat{U}(y_1,y_2)=\begin{pmatrix}\alpha_1(y_1+y_2)-(\beta+1)(y_1+y_2)^{\beta}+\alpha_1 y_1+\alpha_2 y_2-\alpha_1+1\\ \alpha_2(y_1+y_2)-(\beta+1)(y_1+y_2)^{\beta}+\alpha_1 y_1+\alpha_2 y_2-\alpha_2+1\end{pmatrix}$$

$$H(y_1,y_2)=\begin{pmatrix} 2\beta\Big(\frac{\alpha_1}{\beta}-\frac{\beta+1}{2}(y_1+y_2)^{\beta-1}\Big) & \alpha_1+\alpha_2-\beta(\beta+1)(y_1+y_2)^{\beta-1} \\ \alpha_1+\alpha_2-\beta(\beta+1)(y_1+y_2)^{\beta-1} & 2\beta\Big(\frac{\alpha_2}{\beta}-\frac{\beta+1}{2}(y_1+y_2)^{\beta-1}\Big) \end{pmatrix}$$

We have $\nabla\hat{U}(y_1,y_2)=\mathbf{0}$ at the unique point $(y_1^c,y_2^c)=(\frac{\alpha_2-\beta}{\alpha_2-\alpha_1},\frac{\beta-\alpha_1}{\alpha_2-\alpha_1})$ where $y_1^c+y_2^c=1$. At this point

$$\det(H(y_1^c,y_2^c))=-\beta(\beta+1)(\alpha_1+\alpha_2)-(\alpha_1-\alpha_2)^2<0$$

and $\hat{U}(y_1^c,y_2^c)=u_0$. Therefore, the bivariate relief system performance function has a unique critical point which is a saddle point and is outside of the space where a coalition is feasible (i.e., outside of $y_1+y_2>1$). Therefore, the maximum may be the extremes of ∞ and $-\infty$ for y_1 and y_2.

If $\alpha_2>\alpha_1>\beta$ and $\beta<1$, at any point where $y_1+y_2\geq 1$, we have $\nabla\hat{U}(y_1,y_2)>\mathbb{1}$, i.e., the relief system performance function is increasing in y_1 and y_2 at any value of the variables over the decision space. Therefore, the social optimal coalition occurs at (∞,∞). Considering the limits on the number of players, $(y_1^o,y_2^o)=(n_1,n_2)$.

Note that if $\beta<1$ and $\alpha_2<\beta$, $\hat{U}(y_1,n_2)$ is either increasing convex in y_2 at any $y_2\geq 1$ or it eventually becomes increasing convex in y_2. Therefore $(y_1^o,y_2^o)=(n_1,n_2)$ as long as $\hat{U}(n_1,n_2)-\hat{U}(n_1,0)>0$. Defining $\Delta_2(y_1,y_2)=\hat{U}(y_1,y_2)-\hat{U}(0,y_2)$ we have

$$\Delta_2(y_1,y_2)=\alpha_2y_2^2+(1-\alpha_2)y_2+(\alpha_1+\alpha_2)y_1y_2+y_1^{\beta+1}-(y_1+y_2)^{\beta+1}.$$

Note that

$$\frac{\partial\Delta_2(y_1,y_2)}{\partial\alpha_2}=y_2^2+(y_1-1)y_2>0$$
$$\frac{\partial\Delta_2(y_1,y_2)}{\partial\alpha_2\partial y_1}=y_2>0$$

The same holds for $\Delta_1(y_1,y_2)=\hat{U}(y_1,y_2)-\hat{U}(0,y_2)$ when $\alpha_1<\beta$. Therefore, addition of players of type 1 leads to positive values for $\Delta_2(y_1,y_2)$ at lower values of α_2. In other words, there is a threshold τ such that for any $\tau<\alpha_2<\frac{n_2^\beta-1}{n_2-1}$ the social optimal solution includes all n_2 players of type 2 when they are considered together with players of another type, i.e., $y_i^o>y_i^o(0)$. From the proof of Proposition 1a, we can conclude that if

$$\tau<\alpha_2<\frac{n_1^\beta-1}{n_1-1}$$

the social optimal solution includes all players of both types, i.e., $y_2^o=n_2$, while myopic players of type 2 do not join the coalition. i.e., $y_2^*=0$.

When players are forward-looking, without loss of generality we assume $\alpha_2>\alpha_1$. For any given y_2^* we have $U_1(y_1+y_2^*)=\alpha_1-\beta(y_1+y_2^*)^{\beta-1}$. Therefore, at $y_1=0$ we have

$$U_1^{'}(y_1+y_2^*)=\alpha_1-\beta {y_2^*}^{\beta-1}$$

Therefore, when $\beta<1$, the function $U_1(y_1+y_2^*)$ is either increasing convex for all values of y_1 or it starts decreasing convex and later changes to increasing convex. Therefore, as long as $U_1(n_1+y_2^*)>0$ we have $y_1^*=n_1$. We have

$$U_1(n_1+y_2^*)>0\Rightarrow\alpha_1>\frac{(n_1+y_2^*)^\beta-1}{n_1+y_2^*-1}$$

And similarly for α_2 at (n_1, y_2). Therefore we have

$$\begin{cases} y_1^* = 0, y_2^* = 0 & \text{if } \alpha_2 < \dfrac{n_2^\beta - 1}{n_2 - 1} \\ y_1^* = 0, y_2^* = n_2 & \text{if } \alpha_2 > \dfrac{n_2^\beta - 1}{n_2 - 1} > \dfrac{(n_1+n_2)^\beta - 1}{n_1 + n_2 - 1} > \alpha_1 \\ y_1^* = n_1, y_2^* = n_2 & \text{if } \alpha_2 > \alpha_1 > \dfrac{(n_1+n_2)^\beta - 1}{n_1 + n_2 - 1} \end{cases}$$

The social optimal solution includes all n_i players of type i if $\Delta_i(n_1, n_2) > 0$. We note that $\Delta_i(n_1, n_2) > 0$ for $i = 1, 2$ at $\alpha_1 = \alpha_2 = \dfrac{(n_1+n_2)^\beta - 1}{n_1 + n_2 - 1}$. Therefore, and given that $\dfrac{\partial \Delta_i(y_1, y_2)}{\partial \alpha_i} > 0$ the social optimal coalition includes all $n_1 + n_2$ players also at lower values of α_i, $\forall i = 1, 2$. In other words, we have a threshold $\tau < \dfrac{(n_1+n_2)^\beta - 1}{n_1 + n_2 - 1}$ such that if $\tau < \alpha_i < \dfrac{(n_1+n_2)^\beta - 1}{n_1 + n_2 - 1}$ we have $y_i^o = n_i$ and $y_i^* = 0$ for $i = 1, 2$.

Proposition 1b

When $\beta > 1$, if $\alpha_k < \beta$, we have $U_k^{'}(1) < 0$ and $U_k(y_k)$ is decreasing concave for all $y_k > 1$ and therefore HOs of type k do not join any coalition.

On the other hand, if (C.2) holds, i.e., $\alpha_k > \beta$, then $U_k(y_k)$ is concave, initially increasing at $y_k = 1$ and eventually decreasing to $-\infty$. Therefore, $U_k(y_k)$ has two roots on $[1, \infty)$ at $y_k = 1$ and $y_k = y_k^* > 1$. Thus, if $y_k^* < 2$, or equivalently if $U_k(2) < 0$, the only equilibrium is operating alone, i.e., myopic and forward-looking players will decide to not join the coalition. We know that $U_k(2) < 0$ when $\alpha_k < 2^\beta - 1$.

When $\beta > 1$ and $\alpha_k < \beta < 2^\beta - 1$, relief system performance function is decreasing in y_k at any $y_k + y_{3-k} \geq 1$. Therefore, we have $y_k^o = y_k^o(0) = 0$ for $k = 1, 2$. If $\beta < \alpha_k < 2^\beta - 1$, we have $\nabla \hat{U}(y_1, y_2) < \mathbf{0}$ for any coalition where $y_1 + y_2 \geq 2$. Therefore, it is the social optimal that HOs of type k do not join a coalition.

Therefore, when $\beta > 1$ and $\alpha_k < 2^\beta - 1$ the equilibrium and social optimal solutions include no HOs of type k.

We now first consider the case that only type 2 HOs are considered. (The proof for type 1 HOs is similar.) If $\alpha_2 \geq 2^\beta - 1$, we have two possibilities. If

$$1 < \frac{\beta + 1}{2} < \frac{\alpha_2}{\beta} \tag{C.4}$$

then $\hat{U}_{y_2}^{''}(0, 1) > 0$, i.e., social welfare is initially convex increasing, and at

$$y_2 = y_2^{'} = \left(\frac{2\alpha_2}{\beta(\beta+1)}\right)^{\frac{1}{\beta-1}}$$

we have $\hat{U}_{y_2}^{''}(0, y_2) = 0$ and the function shifts from convex increasing to concave increasing. Furthermore,

$$\hat{U}_{y_2}^{'}(0, y_2) = 2\beta\Big(\frac{\alpha_2}{\beta} y - \frac{\beta+1}{2\beta} y^\beta - \frac{\alpha_2 - 1}{2\beta}\Big) \Rightarrow \lim_{y \to \infty} U_{y_2}^{'}(y) = -\infty$$

Therefore, the optimal coalition size is the unique stationary point on $(1, \infty)$, i.e., the optimal coalition size is the unique solution

$$y_2^o = \{y : 2\alpha_2 y - (\beta+1) y^\beta - \alpha_2 + 1 = 0, y > 1\} \tag{C.5}$$

On the other hand, if

$$1 < \frac{\alpha_2}{\beta} < \frac{\beta+1}{2} \tag{C.6}$$

then $\hat{U}''_{y_2}(0,y_2) < 0$ for all $y_2 > 1$, and thus the optimal coalition size is the unique stationary point on $(1,\infty)$, i.e., the optimal coalition size is the unique solution (C.5).

Note that when $\beta > 1$ and $\frac{\alpha_2}{\beta} < \frac{\beta+1}{2}$, we have

$$\hat{U}'_{y_2}(0,2) = 3\alpha_2 + 1 - (\beta+1)2^\beta < 0$$

Therefore, since $\hat{U}'_{y_2}(0,1) > 0$ and $\hat{U}'_{y_2}(0,2) < 0$, the unique stationary point is at a point where $1 < y_2^o < 2$. Therefore, given the integer requirement for y_2^o, the solution is a coalition of maximum 2 HOs, which occurs only when $\alpha_2 \geq 2^\beta - 1$. This condition holds only when $\beta < 2$ and $\frac{\alpha_2}{\beta}$ is very close to $\frac{\beta+1}{2}$.

Further, we have

$$\hat{U}(0,y_2^*) = u_0 + y^* U_2(0,y_2^*) = u_0,$$

i.e., relief system performance at the equilibrium is equal to relief system performance of no coalition (coexistence). Therefore, if $\alpha_2 \geq 2^\beta - 1$, i.e., if the social optimal and equilibrium coalition sizes are greater than 1, concavity of $U_2(0,y_2)$ means

$$\hat{U}(0,y_2) < u_0 \forall y_2 > y_2^*$$

$$\hat{U}(0,y_2) > u_0 \forall y_2 \in (1,y_2^*)$$

which implies that the equilibrium coalition size is greater than the social optimal coalition size, i.e.,

$$y_2^o < y_2^* \tag{C.7}$$

If $n_2 < y_2^*$, the game has two Nash equilibria: (i) no coalition, (ii) a coalition of size n_2. We note that since $U_2(0,y_2)$ is increasing and concave, $U_2(0,n_2) > U_2(0,1) = u_0$. Therefore, the equilibrium of n_2 players is utility-dominant. If we also have $n_2 < y_2^o$, then $y_2^* = y_2^o = n_2$. Otherwise, if $y_2^o < n_2$, then the social optimal coalition includes $y_2^o < y_2^* = n_2$ players. If $y_2^* \leq n_2$, the equilibrium coalition includes $\lfloor y^* \rfloor$ HOs. As shown in (C.7), $y_2^o < y_2^*$.

We now consider both types of players. If $\beta > 1$ and $\alpha_1 > \beta$, $U_1'(1) > 0$, $U_1(y_1)$ is concave and initially increasing at $y_1 = 1$ and eventually decreasing to $-\infty$. Therefore, there exists a $y_1^* > 1$ where $U_1(y_1^*) = 0$. If $n_1 < y_1^*$ then all n_1 players of type 1 join the coalition. Otherwise, the first randomly assigned $\lfloor y_1^* \rfloor$ players of type 1 join the coalition and the remaining will work independently.

First, we consider the case that $n_1 > y_1^*$. Note that (C.1) holds only if $\alpha_2 > \alpha_1$. If $\alpha_2 < \alpha_1$ only $\lfloor y_1^* \rfloor$ players join the coalition. As shown above, the social optimal coalition consists of y_1^o players of type 1 only and the social optimal coalition includes fewer number of players, i.e., $y_1^o < y_1^*$.

If $\alpha_2 > \alpha_1$, players form a coalition including y_1^* and y_2^* players of types 1 and 2, respectively. At this point we have $U_2'(y_1^* + y_2^*) < 0$. We also have

$$\nabla \hat{U}(y_1^*, y_2^*) = \begin{pmatrix} 2\alpha_1 y_1^* + (\alpha_1+\alpha_2)y_2^* - (\beta+1)(y_1^*+y_2^*)^\beta - \alpha_1 + 1 \\ 2\alpha_2 y_2^* + (\alpha_1+\alpha_2)y_1^* - (\beta+1)(y_1^*+y_2^*)^\beta - \alpha_2 + 1 \end{pmatrix}$$

We know that

$$U_2(y_1^* + y_2^*) = \alpha_2(y_1^* + y_2^*) - (y_1^* + y_2^*)^\beta + \alpha_2 - 1 = 0. \tag{C.8}$$

Therefore, we have

$$\hat{U}_{y_1}^{'}(y_1^*, y_2^*) = 2\alpha_1 y_1^* + (\alpha_1 + \alpha_2)y_2^* - \alpha_2(y_1^* + y_2^*) - \beta(y_1^* + y_2^*)^\beta - (\alpha_1 + \alpha_2) + 2$$

Since $U_2^{'}(y_1^* + y_2^*) = \alpha_2 - \beta(y_1^* + y_2^*)^{\beta-1} < 0$, we have

$$\hat{U}_{y_1}^{'}(y_1^*, y_2^*) < 2(\alpha_1 - \alpha_2)y_1^* + (\alpha_1 - \alpha_2)y_2^* + 2 - (\alpha_1 + \alpha_2)$$

When $\alpha_2 > \alpha_1 > \beta > 1$, all of the terms on the right hand side are negative. Therefore, $\hat{U}_{y_1}^{'}(y_1^*, y_2^*) < 0$.

Similarly, we have

$$\begin{aligned}\hat{U}_{y_2}^{'}(y_1^*, y_2^*) &= \alpha_1 y_1^* + \alpha_2 y_2^* - \beta(y_1^* + y_2^*)^\beta - (\alpha_1 + \alpha_2) + 2 \\ &< (\alpha_1 - \alpha_2)y_1^* + 2 - (\alpha_1 + \alpha_2).\end{aligned}$$

Thus, when $\alpha_2 > \alpha_1$ and $\beta > 1$, $\hat{U}_2^{'}(y_1^*, y_2^*) < 0$.

Further, since $U_1^{'}(y_1^*) < 0$ and $U_1^{'}(y)$ is decreasing in y, we have $U_1^{'}(y_1^* + y_2^*) = \alpha_1 - \beta(y_1^* + y_2^*)^{\beta-1} < 0$. Therefore,

$$\hat{U}_{y_1}^{''}(y_1^*, y_2^*) < 2\beta\Big((1 - \frac{\beta+1}{2})(y_1^* + y_2^*)^{\beta-1}\Big).$$

When $\beta > 1$, we have $\hat{U}_{y_1}^{''}(y_1^*, y_2^*) < 0$. Similarly, since $U_2^{'}(y_1^* + y_2^*) < 0$, we have $\hat{U}_{y_2}^{''}(y_1^*, y_2^*) < 0$. Also, as $\hat{U}_{y_1 y_2}^{''}(y_1^*, y_2^*) = \frac{1}{2}(\hat{U}_{y_1}^{''}(y_1^*, y_2^*) + \hat{U}_{y_2}^{''}(y_1^*, y_2^*))$, we also have $\hat{U}_{y_1 y_2}^{''}(y_1^*, y_2^*) < 0$. Therefore, relief system performance function is decreasing at any point (y_1, y_2) where $y_1 \geq y_1^*$ and $y_2 \geq y_2^*$. Therefore, relief system performance is maximized with fewer number of players in the coalition, i.e., $y_1^o + y_2^o < y_1^* + y_2^*$.

If $n_1 < y_1^*$, all n_1 players of type 1 join the coalition. If $n_1 > \left(\frac{\alpha_1}{\beta}\right)^{\frac{1}{\beta-1}}$ then $U_1^{'}(n_1) = \alpha_1 - \beta {n_1}^{\beta-1} < 0$. Therefore, we have $\frac{\alpha_2}{\beta} > {n_1}^{\beta-1}$ only if $\alpha_2 > \alpha_1$. We therefore have $y_1^o + y_2^o < n_1 + y_2^*$. The proof follows the case of $n_1 > y_1^*$.

If $n_1 < y_1^*$ and $n_1 < \left(\frac{\alpha_1}{\beta}\right)^{\frac{1}{\beta-1}}$, it is possible to have $\frac{\alpha_2}{\beta} > {n_1}^{\beta-1}$ for some values of α_2 where $\alpha_2 < \alpha_1$. In that case, from the case of one type of players, we know that the social optimal solution is a coalition of y_1^o HOs of type 1 if $y_1^o < n_1$. Therefore, the optimal solution includes fewer number of HOs, i.e., $y_1^o < n_1 + y_2^*$. If $y_1^o > n_1$, then the social optimal coalition is either a coalition of y_1^o HOs of type 1 or a total of $y_2^o + n_1 < y_1^o$ HOs of the two types. From the case of single player type we know that $y_1^o < y_1^*$. Therefore, the social optimal coalition is always smaller than or equal to the equilibrium outcome.

Proposition 2a

When $\beta < 1$ and players are myopic, similar to the proof of Proposition 1a, we have $y_1^* = n_1$ if $\alpha_1 > 2^\beta - 1$. Then if $U_{D2}(1, n_1) > 0$, n_2 players of type 2 will also join the coalition. We have

$$U_{D2}(1, n_1) > 0 \Rightarrow \alpha_2 > \frac{(\rho n_1 + 1)^\beta - 1}{n_1}$$

Therefore, as the system gets partitioned, i.e., as ρ decreases, the minimum threshold above which players of type 2 will join the coalition also decreases. Therefore, for $\rho < 1$, when we have $\frac{(\rho n_1+1)^\beta - 1}{n_1} < \alpha_2 < \frac{n_1^\beta - 1}{n_1 - 1}$ all players of type 2 will join the coalition while they would not join in a pooled model.

When $\beta < 1$ and players are forward-looking, the proof follows that of Proposition 1a. Without loss of generality, we assume $\alpha_2 > \alpha_1$. We have $y_1^* = n_1$ as long as $U_{D1}(n_1, y_2^*) > 0$. Furthermore,

$$U_{D1}(n_1, y_2^*) > 0 \Rightarrow \alpha_1 > \frac{(n_1 + \rho y_2^*)^\beta - 1}{n_1 + y_2^* - 1}$$

Therefore, we have:

$$\begin{cases} y_1^* = 0, y_2^* = 0 & \text{if } \alpha_2 < \frac{n_2^\beta - 1}{n_2 - 1} \text{ and } \alpha_1 < \frac{(n_1 + \rho n_2)^\beta - 1}{n_1 + n_2 - 1} \\ y_1^* = 0, y_2^* = n_2 & \text{if } \alpha_2 > \frac{n_2^\beta - 1}{n_2 - 1} > \frac{(n_1 + \rho n_2)^\beta - 1}{n_1 + n_2 - 1} > \alpha_1 \\ y_1^* = n_1, y_2^* = n_2 & \text{if } \alpha_2 > \frac{(n_2 + \rho n_1)^\beta - 1}{n_1 + n_2 - 1} \text{ and } \alpha_1 > \frac{(n_1 + \rho n_2)^\beta - 1}{n_1 + n_2 - 1} \end{cases}$$

Note that the thresholds decrease as ρ decreases, i.e., when the system gets partitioned. Therefore, HOs join at lower levels of α_1 and α_2 as compared to a pooled model. Specifically, when $\alpha_2 > \frac{n_2^\beta - 1}{n_2 - 1}$ and $\frac{(n_1+n_2)^\beta - 1}{n_1 + n_2 - 1} > \alpha_1 > \frac{(n_1 + \rho n_2)^\beta - 1}{n_1 + n_2 - 1}$, players of type 1 do not join a pooled coalition, while $y_1^* > 0$ players of type 1 will join a partitioned coalition.

From above, the number of HOs in the coalition remains the same or increases when $\beta < 1$. Note that

$$\frac{\partial \hat{U}_D(y_1, y_2)}{\partial \rho} = -\beta y_1 y_2 \left((y_1 + \rho y_2)^{\beta-1} + (y_2 + \rho y_1)^{\beta-1} \right) < 0 \tag{C.9}$$

Thus, when the number of HOs remains the same, relief systsem performance is higher in a partitioned model, i.e., when ρ is smaller. When HOs are forward-looking and the equilibrium partitioned coalition is larger than the equilibrium pooled coalition (i.e., when $\alpha_2 > \frac{n_2^\beta - 1}{n_2 - 1}$ and $\frac{(n_1+n_2)^\beta - 1}{n_1 + n_2 - 1} > \alpha_1 > \frac{(n_1 + \rho n_2)^\beta - 1}{n_1 + n_2 - 1}$), the improvement in relief system performance due to partitioning (i.e., the difference between $\hat{U}_D(y_1^*, y_2^*)$ at ρ and $\rho = 1$) equals

$$n_1 \left(\alpha_1 (n_1 - 1) - (n_1 + \rho n_2)^\beta + 1 \right) + n_2 \left(n_1 (\alpha_1 + \alpha_2) + n_2^\beta - (n_2 + \rho n_1)^\beta \right).$$

This value is always positive when $\alpha_2 > \frac{n_2^\beta - 1}{n_2 - 1} > \frac{(n_2 + \rho n_1)^\beta - 1}{n_1 + n_2 - 1}$ and $\alpha_1 > \frac{(n_1 + \rho n_2)^\beta - 1}{n_1 + n_2 - 1}$.

When $\beta > 1$ and players are myopic, $y_1^* > 0$ players of type 1 join as long as $\alpha_1 > \beta$. We note that

$$\frac{\partial U_{Dk}(y_k, y_{3-k})}{\partial \rho} = -y_{3-k} \beta (y_k + \rho y_{3-k})^{\beta - 1} < 0 \qquad \forall k = 1, 2. \tag{C.10}$$

As ρ decreases, the value of $U_{Dk}(y_k, y_{3-k})$ increases at any given point. Therefore, the value of y^* increases as the system is partitioned.

Then, $y_2^* > 0$ will also join if $U_{D2}(0, y_1^*) > 0$. We have

$$U_{D2}(0, y_1^*) = U_{D1}(y_1^*, 0) + (\alpha_2 - \alpha_1)(y_1^* - 1) + (1 - \rho^\beta) {y_1^*}^\beta$$

Noting that $U_{D1}(y_1^*, 0) = 0$, as ρ decreases, $U_{D2}(0, y_1^*) > 0$ at lower values of α_2. From (C.10) we know that the value of $U_{D2}(y_1^*, y_2)$ increases as ρ is decreased. Further, at any y_2 we have

$$\frac{\partial^2 U_{D2}(y_1^*, y_2)}{\partial \rho \partial y_1^*} = -\beta (y_2 + \rho y_1^*)^{\beta - 1} - y_1^* \beta (\beta - 1) \rho (y_2 + \rho y_1^*)^{\beta - 2} < 0$$

Therefore, the increase in $U_{D2}(y_1^*, y_2)$ due to partitioning is higher at higher values of y_1^*. Similarly, for $k = 1, 2$ we have

$$\begin{aligned}\frac{\partial^2 \hat{U}_D(y_k, y_{3-k})}{\partial y_k \partial \rho} =& - y_{3-k}\beta(y_k + \rho y_{3-k})^{\beta-1} - y_{3-k}y_k\beta(\beta-1)(y_k + \rho y_{3-k})^{\beta-2} \\ & - y_{3-k}\beta(y_{3-k} + \rho y_k)^{\beta-1} - y_{3-k}y_k\beta\rho(y_{3-k} + \rho y_k)^{\beta-2} < 0 \end{aligned} \tag{C.11}$$

$$\frac{\partial^3 \hat{U}_D(y_k, y_{3-k})}{\partial y_k \partial y_{3-k} \partial \rho} = - \beta\left(\beta + (\beta-1)^2\rho^{\beta-2} + \beta\rho^{\beta-1}\right) < 0 \tag{C.12}$$

Therefore, when $\beta > 1$, partitioning leads to a better relief system performance at any given point (y_1, y_2). The improvement in relief system performance due to partitioning further increases when the number of coordinating HOs increases. Therefore, since the number of coordinating HOs at the equilibrium increases by partitioning, relief system performance at the equilibrium also increases.

Proposition 2b

When $\beta < 1$, following the proof of Proposition 1a, we define $\Delta_1(y_1, y_2) = \hat{U}(y_1, y_2) - \hat{U}(0, y_2)$. We have

$$\Delta_1(y_1, y_2) = \alpha_1 y_1^2 + y_2^{\beta+1}(1-\alpha_1)y_1 + (\alpha_1 + \alpha_2)y_1y_2 - y_1(y_1 + \rho y_2)^\beta - y_2(y_2 + \rho y_1)^\beta$$

Note that

$$\frac{\partial \Delta_1(y_1, y_2)}{\partial \rho} = -y_1y_2\left((y_1 + \rho y_2)^{\beta-1} + (y_2 + \rho y_1)^{\beta-1}\right) < 0$$

Therefore, as ρ decreases, we have $\Delta_1(y_1, y_2) > 0$ and thus $y_1^o = n_1$ at lower values of α_1. The same holds for $\Delta_2(y_1, y_2)$ and α_2.

As shown in (C.9), relief system performance at any point increases as the system gets partitioned. Further, from (C.11) and (C.12), for any $\beta > 0$, the slope of relief system performance function increases at any point when ρ is decreased. Thus, as the system gets partitioned, the optimal coalition size and the optimal relief system performance both increase.

Proposition 3

Low time burden ($\beta < 1$)

We define α_k^o and α_k^* as the value of α_k above which the social optimal and the equilibrium includes n_k players of type k. We have

$$\begin{aligned}\alpha_k^o =& \frac{n_k(n_k + \rho n_{3-k})^\beta + n_{3-k}(n_{3-k} + \rho n_k)^\beta - \alpha_{3-k}n_kn_{3-k} - n_{3-k}^{\beta+1}}{n_k^2 + n_kn_{3-k} - n_k}, \\ \alpha_k^* =& \frac{(n_k + \rho n_{3-k})^\beta - 1}{n_k + n_{3-k} - 1}.\end{aligned}$$

Further,

$$\frac{\partial \alpha_k^o}{\partial \rho} = \frac{n_{3-k\beta}}{n_k + n_{3-k} - 1}\left((n_k + \rho n_{3-k})^{\beta-1} + (n_{3-k} + \rho n_k)^{\beta-1}\right)\frac{\partial \alpha_k^*}{\partial \rho} = \qquad \frac{n_{3-k}\beta(n_k + \rho n_{3-k})^{\beta-1} - 1}{n_k + n_{3-k} - 1}.$$

We have $\frac{\partial \alpha_k^o}{\partial \rho} > \frac{\partial \alpha_k^*}{\partial \rho}$. Therefore, as ρ is decreased, the range of α_k for which the social optimal includes n_k players increases more than the range for which the equilibrium coalition includes n_k players. In other words, when the system gets partitioned, there is a range of α_k for which the social optimal includes all n_k HOs of type k, while the equilibrium remains the same as a pooled coalition where players of type k do not join.

High time burden ($\beta > 1$) From Proposition 1b we know that when $\rho = 1$, we have $\frac{\partial \hat{U}(y_1^*, y_2^*)}{\partial y_k} < 0$ for $k = 1, 2$. From Proposition 2a, we know that y_k^* increases or remains the same as the system gets partitioned. Note that at y_k^* we have $U_{Dk}(y_1^*, y_2^*) = 0$ and at y_k^o we have $\frac{\partial \hat{U}_D(y_1^o, y_2^o)}{\partial y_k} = 0$. From (C.10) and (C.11) we have $\frac{\partial^2 \hat{U}_D(y_k, y_{3-k})}{\partial y_k \partial \rho} < \frac{\partial U_{Dk}(y_k, y_{3-k})}{\partial \rho} < 0$. Therefore, for a certain decrease in ρ, the optimal coalition size increases by a larger value than the equilibrium coalition size increases. Therefore, when the system gets partitioned the difference between the optimal relief system performance and its value at the equilibrium decreases.

Note that we have

$$\frac{\partial \hat{U}(y_1, y_2)}{\partial y_k} = (2y_k + y_{3-k} - 1)\alpha_k - (y_k + \rho y_{3-k})^{\beta} - \beta\Big(y_k(y_k + \rho y_{3-k})^{\beta-1} + \rho y_{3-k}(y_{3-k} + \rho y_k)^{\beta-1}\Big) + \alpha_{3-k} y_{3-k} + 1$$

Therefore,

$$\lim_{\rho \to 0^+} \frac{\partial \hat{U}(y_1, y_2)}{\partial y_k} = (2y_k + y_{3-k} - 1)\alpha_k - (\beta + 1) y_k^{\beta} + \alpha_{3-k} y_{3-k} + 1$$

Note that

$$\lim_{\rho \to 0^+} U_{Dk}(y_k^*, y_{3-k}^*) = (2y_k + y_{3-k} - 1)\alpha_k - y_k^{\beta} + 1 = 0$$

Therefore, we have

$$\lim_{\rho \to 0^+} \frac{\partial \hat{U}(y_1^*, y_2^*)}{\partial y_k} = \alpha_{3-k} y_{3-k}^* - (\beta {y_k^*}^{\beta} - \alpha_k y_k^*).$$

At high levels of α_{3-k}, it is possible to have $y_k^* < y_k^o$ if the level of partitioning is very high. In other words, under such circumstances, there is a $0 < \rho < 1$ for which we have $y_k^* = y_k^o$.

D. Numerical experiments

D.1. Robustness to the logarithmic assumption

Evidence from practice suggests a concave relationship between coalition size and response delays. In our analytical model, we adopt a natural logarithmic form for this relationship to enable closed-form solutions. To evaluate the robustness of our findings to this functional form, we conduct a numerical simulation using alternative concave delay functions.

Specifically, we model the total response time for a player in a coalition of size y as $T + t_B y^{\gamma}$ for $\gamma \in (0, 1)$. This functional form allows us to vary the degree of concavity, with smaller values of γ representing more efficient coordination mechanisms (i.e., marginal time costs increase less rapidly) and larger values approximating less efficient coalitions with a more linear time increase. The resulting timeliness disutility function becomes: $e^{b(T + t_B y^{\gamma})} - e^{bT} = e^{bT}(e^{\beta y^{\gamma}} - 1)$.

We simulate the model across a wide range of input parameters to assess whether the core insights from our theoretical results continue to hold. Specifically, we test five values of γ: 0.2, 0.4, 0.5, 0.6, and 0.8. Table D.1 summarizes the other parameter values used in the simulation. For each value of γ, a total of 1,849,600 instances are generated.

Table D.1 Parameter values used in the numerical experiment 1

Parameter	Minimum	Maximum	Increment
β	0.05	1.95	0.1
ρ	0.2	1	0.2
n_1	4	32	4
n_2	4	32	4
α_1	0.25	2.65	0.15
α_2	0.25	2.65	0.15

we generate 1,849,600 instances. Of these, 369,920 simulate pooled coalitions ($\rho = 1$), while the remaining 1,479,680 represent varying degrees of partitioned coalitions.

Table D.2 and Figure D.1 present the simulation results. Overall, we find strong support for the robustness of our main findings. Across all parameter combinations, the average satisfaction rate across the Propositions is 92.98%. In particular, Propositions 2a and 2b hold in 99.05% of cases, while Proposition 3 is satisfied in 90.85% of instances, indicating that the main insights regarding the advantages of partitioning are not sensitive to the logarithmic delay assumption.

Proposition 1b, which warns against the risk of overly large coalitions in high-urgency settings, shows a 93.91% satisfaction rate. This Proposition holds in all simulated instances for higher values of γ, where response delays increase more steeply with coalition size. At lower values, particularly $\gamma = 0.2$, we observe reduced satisfaction, which is consistent with our theoretical insights–specifically, that more efficient coordination mechanisms can help mitigate the risks of overly large coalitions.

Finally, Proposition 1a exhibits a complementary pattern. It is more frequently satisfied at lower values of γ, and shows reduced satisfaction under more inefficient coordination conditions (higher γ). On average across all simulations, this Proposition holds in 82.05% of cases, indicating a moderate sensitivity that reinforces its underlying trade-offs.

Table D.2 Proposition satisfaction across gamma values

**Gamma**	**Proposition 1 (Total: 1,849,600)**		**Proposition 2 (Total: 7,398,400)**		**Proposition 3 (Total: 7,398,400)**
	P1a (%)	P1b (%)	P2a (%)	P2b (%)	P3 (%)
0.2	100.00	71.17	100.00	100.00	95.81
0.4	95.74	98.43	99.04	100.00	87.64
0.5	78.06	99.96	97.97	100.00	88.42
0.6	70.85	100.00	97.12	100.00	90.22
0.8	65.59	100.00	96.34	100.00	92.14
Overall	82.05	93.91	98.09	100.00	90.85

D.2. Proposition 2a

In Proposition 2a, we find that, when $\beta < 1$ and HOs are myopic, it is possible for the partitioned model to lead to a worse relief system performance as compared to a pooled model. We investigated the prevalence of this phenomenon in a set of numerical experiments. We simulated the pooled and partitioned models, varying the values for parameters

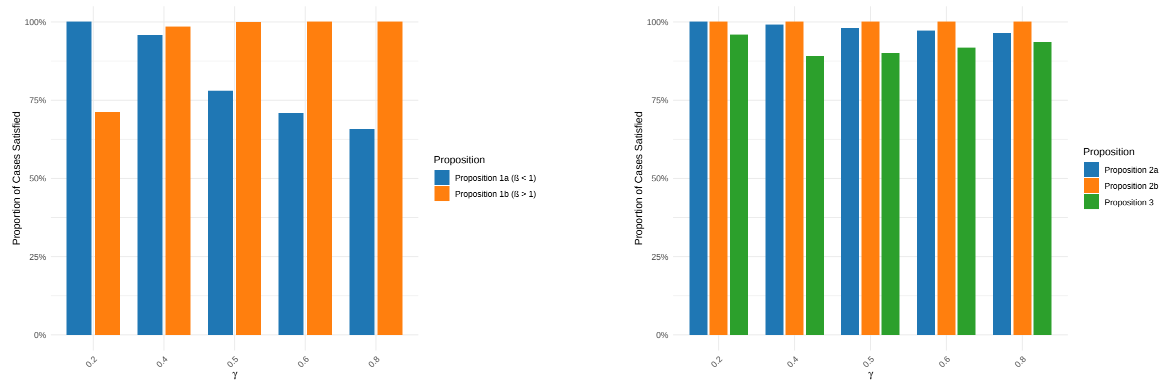

(a) Propositions 1a and 1b (b) Propositions 2a, 2b, and 3

Figure D.1 Simulation results – satisfaction rates of Propositions by γ.

ρ, n_1, n_2, α_1, and α_2, and compared the solutions of the partitioned model with a given β and those of a pooled model where $\beta = 1$. Table D.3 indicates the values used for all of the parameters.

Table D.3 Parameter values used in the numerical experiment 2

Parameter	Minimum	Maximum	Increment
β	0.05	0.95	0.1
ρ	0.05	0.95	0.1
n_1	4	32	4
n_2	4	32	4
α_1	0.25	1.45	0.15
α_2	0.25	1.45	0.15

Table D.4 summarizes the results and compares three categories of outcomes in terms of different parameters. Among 518,400 simulated examples, the partitioned model has a better relief system performance than the pooled model in 395,457 cases (76.28%). In 122,312 examples (23.59%), the solutions of the partitioned and the pooled models are identical where at most one type of HOs join the coalition and therefore both models result in the same relief system performance. It is only in 631 cases (0.12%) that the partitioned model has a relief system performance worse than that of the pooled model. Notably, these cases occur only when the time burden of coordination is relatively high, i.e., close to 1. The minimum value of β in cases where partitioning results in a worse relief system performance equals 0.75. Further, we observe that the decline in relief system performance only happens when large HOs are first in the sequence and they are fewer in number than small HOs. The minimum (average) value observed for $\frac{\alpha_1}{\alpha_2}$ equals 1.43 (3.40) when partitioning leads to worse relief system performance and 0.17 (1.45) when the opposite holds. The minimum (average) ratio between the number of small HOs and large HOs ($\frac{n_2}{n_1}$) equals 1.5 (5.23) in numerical examples where the partitioned model leads to a worse relief system performance than the pooled model and it equals 0.13 (1.51) where relief system performance is better in the partitioned model. Figure D.2 shows the distribution of

these ratios in cases where the partitioned model leads to worse or better relief system performance than the pooled model.

Table D.4 Summary statistics by scenario with best relief system performance

Metric	Statistic	Partitioned	Equal	Pooled	Overall
**Number of observations**	–	395,457	122,312	631	518,400
**Time burden** (β)	Minimum	0.05	0.35	0.75	0.05
	Maximum	0.95	0.95	0.95	0.95
	Mean	0.42	0.76	0.91	0.50
	Median	0.35	0.75	0.95	0.50
**Relative capacity** (α_1/α_2)	Minimum	0.17	0.17	1.43	0.17
	Maximum	5.80	5.80	5.80	5.80
	Mean	1.45	1.00	3.40	1.35
	Median	1.12	0.57	3.25	1.00
**Relative size** (n_2/n_1)	Minimum	0.13	0.13	1.50	0.13
	Maximum	8.00	8.00	8.00	8.00
	Mean	1.51	1.56	5.23	1.53
	Median	1.00	1.00	5.00	1.00

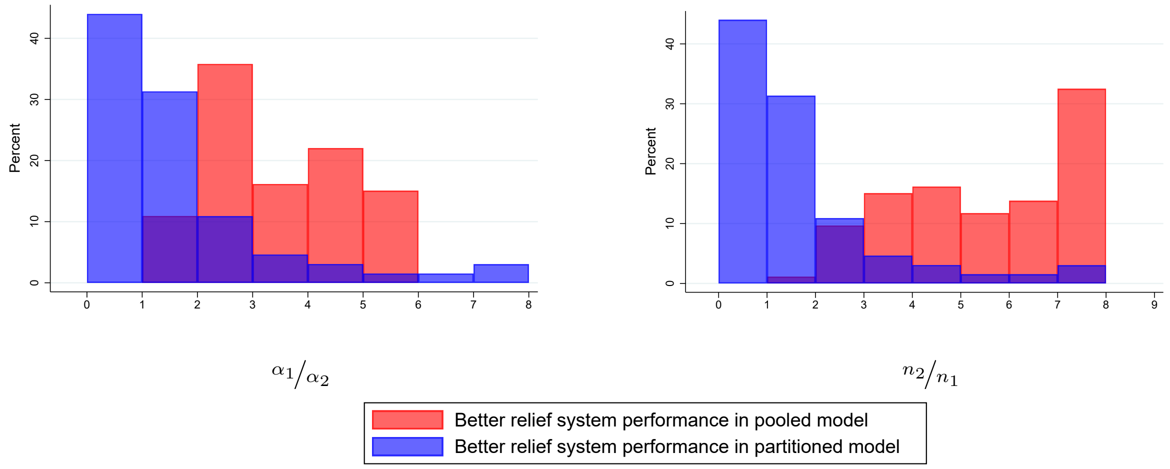

Figure D.2 Distributions of $\frac{\alpha_1}{\alpha_2}$ and $\frac{n_2}{n_1}$ in the numerical experiments for myopic players and low time burden in examples where relief system performance in pooled and partitioned models are different.
